# Crystal structures of three new *N*-halo­methyl­ated quaternary ammonium salts

**DOI:** 10.1107/S2056989015017181

**Published:** 2015-09-26

**Authors:** Carolina Múnera-Orozco, Rogelio Ocampo-Cardona, David L. Cedeño, Rubén A. Toscano, Luz Amalia Ríos-Vásquez

**Affiliations:** a Departamento de Química, Universidad de Caldas, Manizales, Caldas, Colombia; b Department of Chemistry, Illinois State University, Normal, Illinois, USA; c Instituto de Química, UNAM, Circuito Exterior, Ciudad Universitaria, Delegación Coyoacán, C.P. 04510, México, D.F., Mexico

**Keywords:** crystal structure, *N*-halo­methyl­ated quaternary ammonium salts, cation–π inter­action, halogen bond, hydrogen bonding

## Abstract

In the crystals of the title *N*-halo­methyl­ated quaternary ammonium salts, C_19_H_23_IN^+^·I^−^, (I) [systematic name: *N*-(4,4-di­phenyl­but-3-en-1-yl)-*N*-iodo­methyl-*N*,*N*-di­methyl­ammonium iodide], C_20_H_25_IN^+^·I^−^, (II) [systematic name: *N*-(5,5-di­phenyl­pent-4-en-1-yl)-*N*-iodo­methyl-*N*,*N*-di­methyl­ammonium iodide], and C_21_H_27_IN^+^·I^−^, (III) [systematic name: *N*-(6,6-di­phenyl­hex-5-en-1-yl)-*N*-iodo­methyl-*N*,*N*-di­methyl­ammonium iodide], there are short I⋯I^−^ inter­actions of 3.564 (4), 3.506 (1) and 3.557 (1) Å for compounds (I), (II) and (III), respectively. Compound (I) crystallizes in the Sohncke group *P*2_1_ as an ‘enanti­opure’ compound and is therefore a potential material for NLO properties. In the crystal of compound (I), mol­ecules are linked by C—H⋯I^−^ and C—H⋯π inter­actions which, together with the I⋯I^−^ inter­actions, lead to the formation of ribbons along [100]. In (II), there are only C—H⋯I^−^ inter­actions which, together with the I⋯I^−^ inter­actions, lead to the formation of helices along [010]. In (III), apart from the I⋯I^−^ inter­actions, there are no significant inter­molecular inter­actions present.

## Abstract 2

In the crystals of the title *N*-halo­methyl­ated quaternary ammonium salts, there are short I⋯I^−^ inter­actions of 3.564 (4), 3.506 (1) and 3.55781) Å for compounds (I), (II) and (III), respectively. In (I), mol­ecules are linked by C—H⋯I^−^ and C—H⋯π inter­actions, together with the I⋯I^−^ short contacts, forming ribbons along [100]. In (II), there are only C—H⋯I^−^ inter­actions, which together with the I⋯I^−^ short contacts, lead to the formation of helices along [010]. In (III), apart from the I⋯I^−^ short contacts, there are no other significant inter­molecular inter­actions present.

## Chemical context and background to halogen bonding and cation–π inter­actions   

Quaternary ammonium salts have been widely studied as anti-cancer (Wang *et al.*, 2012 ▸; Song *et al.*, 2013 ▸), anti-fungal (Ng *et al.*, 2006 ▸), anti-HIV-1 (Shiraishi *et al.*, 2000 ▸), anti-bacterial (Calvani *et al.*, 1998 ▸), anti-malarial (Calas *et al.*, 1997 ▸; Calas *et al.*, 2000 ▸) and anti-leishmanial (Mavromoustakos *et al.*, 2001 ▸) pharmaceuticals. Our research group has been working in the past few years on the activity of quaternary *N*-halomethyl ammonium salts for likely pharmaceutical purposes, specifically against axenic *L. (V) panamensis* and *L. (L) amazonensis* parasites, human pathogenic species that cause cutaneous and mucocutaneous leishmaniasis. The experiments proved that these compounds are very promising anti-leishmanial mol­ecules, and very significant changes in their activity were observed upon a slight modification of the carbon skeleton by only a single methyl­ene unit (Ríos-Vásquez *et al.*, 2015 ▸). A preliminary effort at understanding a structure–activity relationship with three *N*-iodo­methyl quaternary ammonium salts (I), (II) and (III) of the form [ICH_2_N(CH_3_)_3_(CH_2_)_*n*_CH=C(Ph)_2_]^+^·I^−^ (with *n* = 2, 3 and 4, respectively) is currently being carried out. One possible approach to understand the different activities is to establish what kind of inter­actions are present in compounds (I)–(III), for example whether C—I⋯I^−^ (Desiraju *et al.*, 2013 ▸), C—H⋯I^−^ (Glidewell *et al.*, 1994 ▸), C—H⋯π (Nishio *et al.*, 1998 ▸) or cation–π (Dougherty, 1996 ▸), and if so, how these inter­actions may affect their structure and biological properties.

As defined by Inter­national Union for Pure and Applied Chemistry (IUPAC): *a halogen bond occurs when there is evidence of a net attractive inter­action between an electrophilic region associated with a halogen atom in a mol­ecular entity and a nucleophilic region in another, or the same, mol­ecular entity* (Desiraju *et al.*, 2013 ▸). Halogen bonds are characterized by *X*⋯*X* distances that are clearly shorter than the van der Waals radii sum (Formigué, 2009 ▸; Awwadi *et al.*; 2006 ▸); otherwise this inter­action is neglected. In a similar way, the existence of C—H⋯*X* hydrogen bonds (*X* = F, Cl, Br or I) in neutral organic mol­ecules (Aakeröy & Seddon, 1993 ▸) and even in organic salts has been recognized. On the other hand, a special kind of hydrogen bond, defined as a weak inter­action between a soft acid (*i.e.* an *sp* ^3^, *sp* ^2^ or *sp* C—H system) and a soft base (*i.e.* an aromatic, olefinic or acetilenic *p* system), with a significant role on diverse chemical and biological phenomena has recently been described (Nishio, 2012 ▸). In particular, this inter­action exerts an observable influence on host–guest recognition and crystal packing in the solid state. A related attraction is the cation–π inter­action, which is regarded as an electrostatic attraction between a positive charge and the quadrupole moment of an aromatic ring (Dougherty, 1996 ▸). A cation–π inter­action between aromatic and ammonium ions is known to play an important role in many biological systems (Ma & Dougherty, 1997 ▸; Dougherty, 2013 ▸; Sussman *et al.*, 1991 ▸; Chen *et al.*, 2011 ▸). Part of our research inter­est is focused not only in understanding the reactive nature of alpha ammonium distonic radical cations which are generated from *N*-halo­methyl­ated quaternary ammonium salts (Ríos *et al.*, 1996 ▸; Ríos, Bartberger *et al.*, 1997 ▸), but also in trying to understand how these salts behave against *Leishmania* parasites (Ríos-Vásquez *et al.*, 2015 ▸). The recognition of the occurrence of some supra­molecular inter­actions in these salts may lead to a better understanding of the likely novel biological binding sites, and therefore to new suggestions about biocatalytic mechanisms.
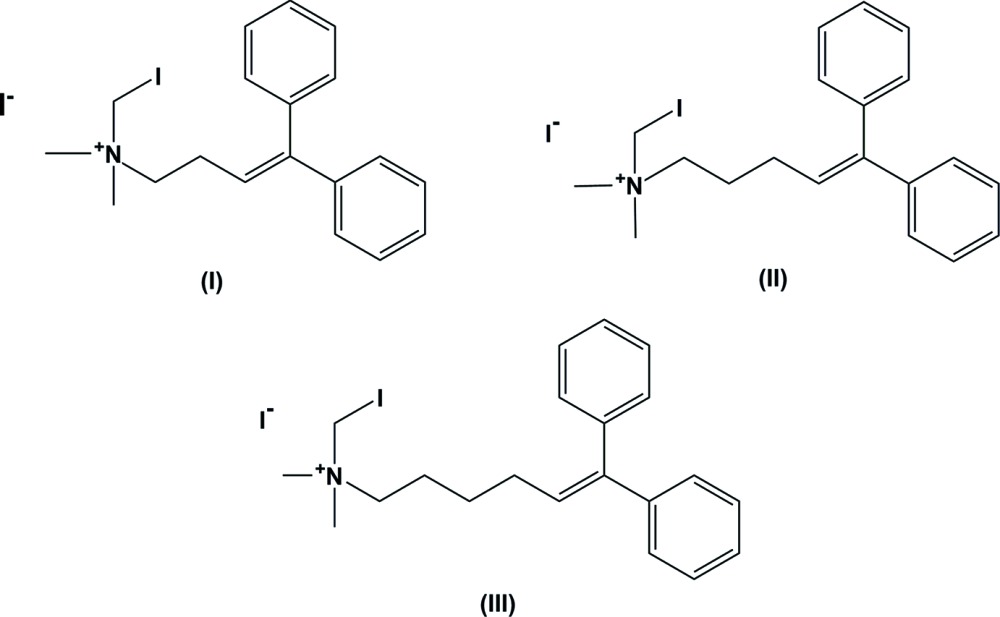


The title *N*-iodo­methyl quaternary ammonium salts, (I)–(III), were synthesized following standard procedures used for other related compounds (Newcomb *et al.*, 1993 ▸; Horner *et al.*, 1995 ▸) and suitable crystals were obtained (Múnera-Orozco, 2014 ▸). This paper reports a comparative crystal structure and supra­molecular inter­actions analysis for the aforementioned compounds.

## Structural commentary   

**Figure 1:**
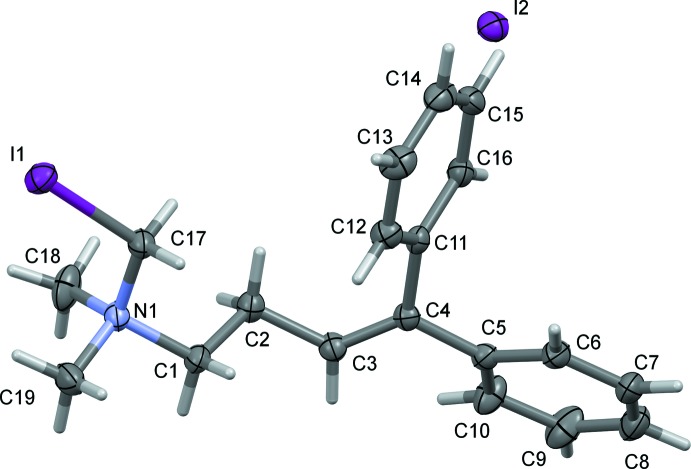
The mol­ecular structure of compound (I), showing the atom labelling. Displacement ellipsoids are drawn at the 50% probability level.

Compound (I), Fig. 1 ▸, crystallizes in the non-centrosymmetric monoclinic space group *P*2_1_ and is therefore, a potential material for NLO properties. The asymmetric unit consists of an ammonium cation and an iodide anion. In the geminal-substituted di­phenyl­ethene unit, the phenyl rings (C5–C10 and C11–C16) are inclined to one another by 74.6 (2)°, and are twisted from the mean plane of the central C=C bond fragment (C2–C5/C11) by 33.2 (2) and 61.4 (2)°, respectively. Co-planarity of the olefin skeleton and the peripheral phenyl rings is prevented because of steric congestion between the associated phenyl rings. The conformation of the side chain reveals an *all-trans* extended conformation with the iodo­methyl moiety on one side of the backbone chain, with bond lengths and angles in the expected ranges.

**Figure 2:**
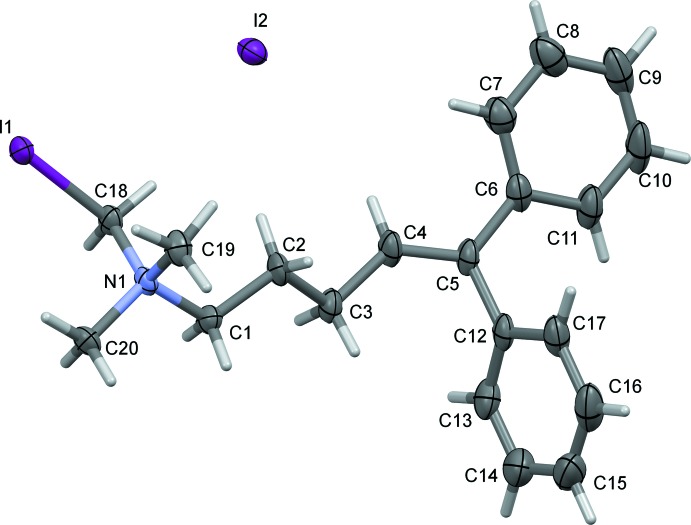
The mol­ecular structure of compound (II), showing the atom labelling. Displacement ellipsoids are drawn at the 50% probability level.

In compound (II), Fig. 2 ▸, the dihedral angles between the mean planes of the C=C double-bond fragment (C3–C6/C12) and the two phenyl rings (C6–C11 and C12–C17) are 31.1 (4) and 58.6 (4)°, respectively, while the phenyl rings are inclined to one another by 76.2 (4)°. The *N*-iodo­methyl-*N*,*N*-dimethyl-*N*-propyl­ammonium moiety adopts a fully extended conformation with one methyl group and the iodo­methyl unit on opposite sides of the backbone of the side chain (Fig. 2 ▸). This conformation seems to be partially supported by a C—H⋯I^−^ hydrogen bond (Table 2 and *Supra­molecular features*).

**Figure 3:**
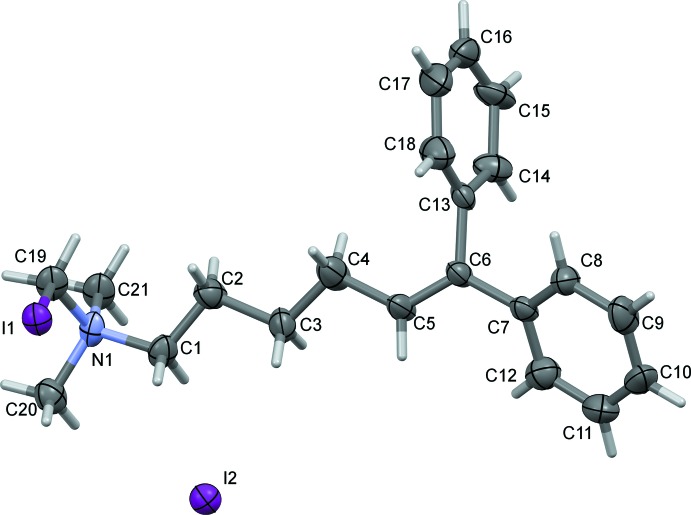
The mol­ecular structure of compound (III), showing the atom labelling. Displacement ellipsoids are drawn at the 50% probability level.

In compound (III), Fig. 3 ▸, the phenyl rings are twisted out of the plane defined by the ethyl­ene moiety (C4–C7/C13), making dihedral angles of 38.7 (4) and 78.7 (6)° for the *trans* (C7–C12) and *cis* (C13–C18) phenyl rings, respectively. The phenyl rings are inclined to one another by 78.5 (6)°. The alkyl­amino side chain is almost fully extended away from the geminal-substituted ethene group.

## Supra­molecular features   

**Figure 4:**
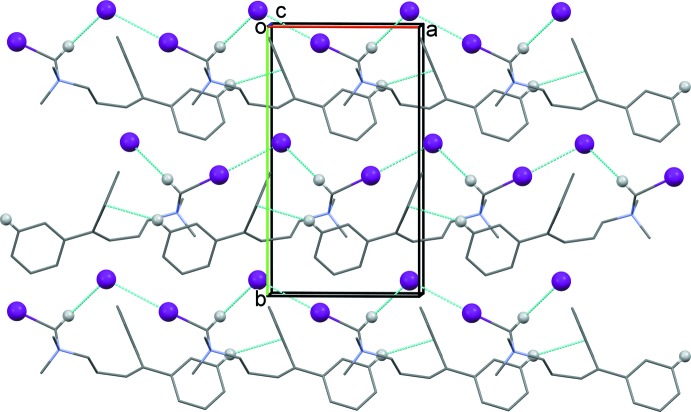
The crystal packing of compound (I), viewed along the *b* axis, showing the inter­molecular contacts (dashed lines; see Table 1 ▸).

In the crystal of (I), ribbons are formed, by I1⋯I2^i^ contacts [3.564 (4) Å; symmetry code: (i) −*x* − 1, *y* − 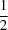, −*z* + 1] and C—H⋯I^−^ hydrogen bonds, along the *a-*axis direction. The chains are reinforced by C—H⋯π inter­actions (Fig. 4 ▸ and Table 1 ▸).

**Figure 5:**
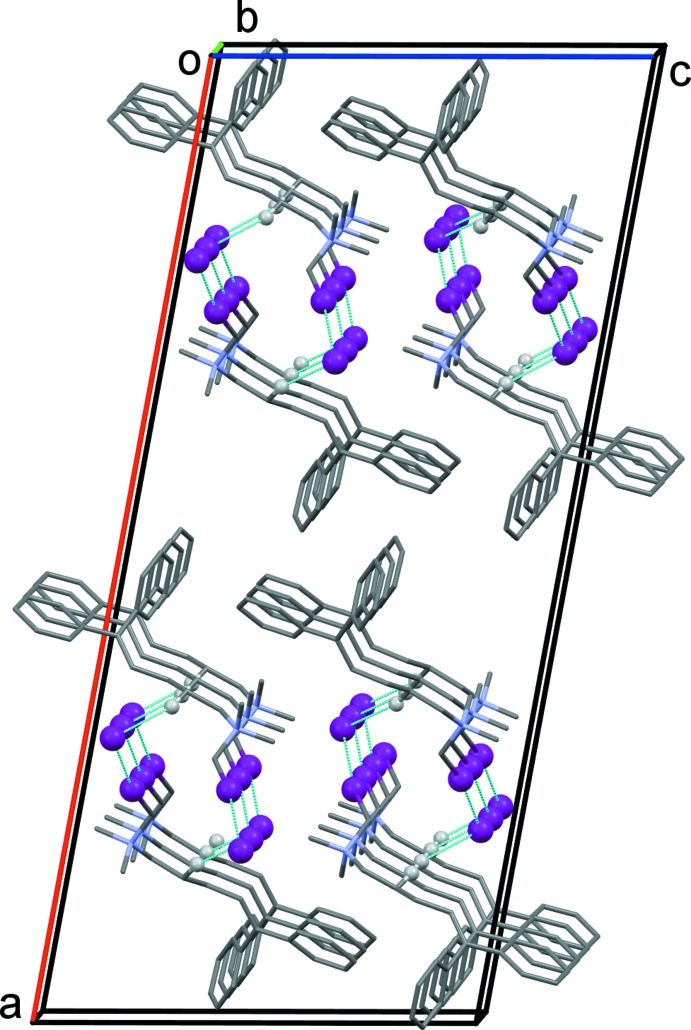
The crystal packing of compound (II), viewed along the *b* axis, showing the inter­molecular contacts (dashed lines; see Table 2 ▸).

In the crystal of (II), helical chains along the *b-*axis direction are formed by mol­ecules linked *via* C—H⋯I^−^ (Table 2 ▸) and I1⋯I2^ii^ inter­actions [3.506 (1) Å; symmetry code: (ii) −*x* + 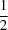, *y* − 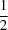, −*z* + 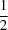]; as shown in Fig. 5 ▸. Here no C—H⋯π inter­actions are present in the crystal packing. The closest distance between the ammonium substituents and any of the phenyl rings is *ca* 7.18 Å. These features clearly rule out an intra­molecular cation–π inter­action for this mol­ecule in the solid state. However, in studies of distonic radical cation (Ríos *et al.* 1996 ▸; Yates *et al.*, 1986 ▸), evidence is presented that the active conformation of the alkyl­amino side chain is oriented toward and above the plane of the C=C double bond of the geminal-substituted ethene group. These results confirm that there is considerable freedom of rotation about the bonds separating the basic amino function and the tricyclic system, and thus numerous inter­convertible side-chain conformations, differing only slightly in potential energy, may exist.

**Figure 6:**
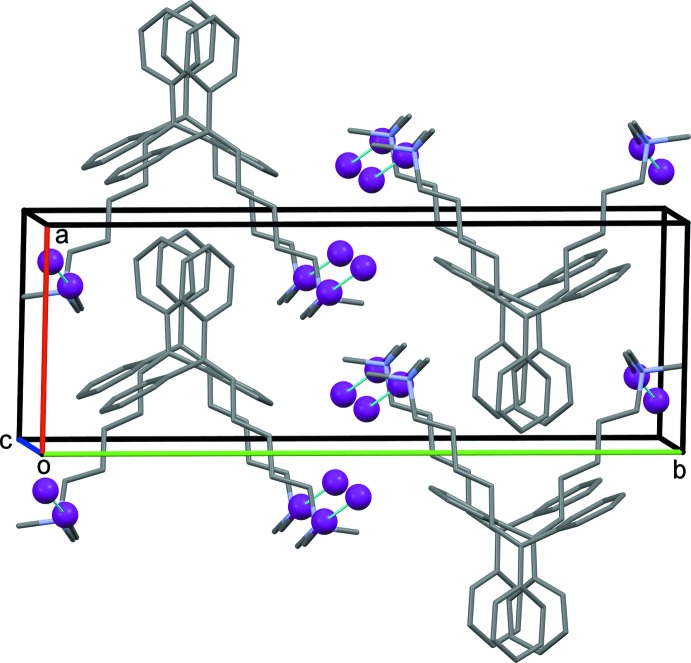
The crystal packing of compound (III), viewed along the *b* axis.

In the crystal of (III), apart from the I1⋯I2^iii^ contact of 3.557 (1) Å [symmetry code: (iii) −*x*, −*y* + 1, −*z*], there are no other significant inter­molecular contacts present (Fig. 6 ▸). The only possible conclusion regarding the crystal structure of (III) is that the steric requirements in this mol­ecule outweigh the additional stabilization obtained by an intra­molecular cation–π inter­action.

## Synthesis and crystallization   

**Figure 7:**
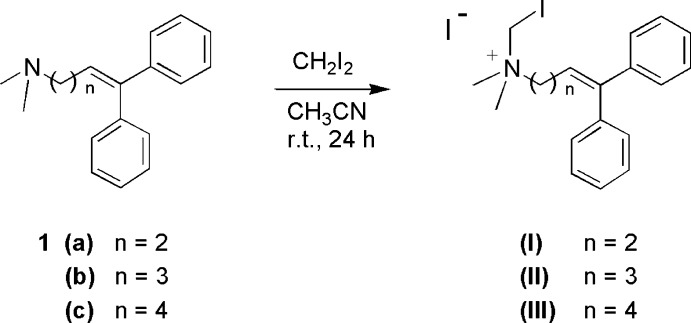
The general procedure for the preparation of the title quaternary ammonium salts.

The general procedure for the preparation of the title quaternary ammonium salts is illustrated in Fig. 7 ▸ for compounds (I)–(III). The reactions were carried out following a standard literature method (Ríos *et al.*, 1996 ▸) starting from the appropriate amine [*N*,*N*-dimethyl-4,4-di­phenyl­but-3-en-1-amine 1(a), *N*,*N*-dimethyl-5,5-di­phenyl­pent-4-en-1-amine 1(b) and *N*,*N*-dimethyl-6,6-di­phenyl­hex-5-en-1-amine 1(c)]. Typically, CH_2_I_2_ (4 eq) and 1 eq of the starting tertiary amine [for example, compound 1(a) for the synthesis of (I); as shown in Fig. 7 ▸] were dissolved in aceto­nitrile. The reactions were allowed to run overnight at room temperature, and the precipitated salts were filtered off and washed several times with diethyl ether, and then recrystallized from a binary mixture water–iso­propanol. The desired products were obtained as colourless crystals.

**Compound (I)**: The product was obtained as a white solid in 74% yield; m.p. 425–427 K. ^1^H NMR (DMSO, 300 MHz, δ, p.p.m.): 2.49 (*m*, 2H), 3.12 (*s*, 6H), 3.50 (*m*, 2H), 5.05 (*s*, 2H), 6.07 (*t*, *J* = 7.4 Hz, 1H), 7.15–7.58 (*m*, 10H) p.p.m. ^13^C NMR (DMSO, 75 MHz,, p.p.m.) 23.70, 31.49, 51.66, 63.58, 121.92, 127.19–129.51, 138.79, 141.62, 145.03 p.p.m. Elemental analysis calculated for C_19_H_23_NI_2_: C, 43.95%; H, 4.46%; N, 2.70%; found, C, 43.48%; H, 4.35%; N, 2.68%. MS–ESI calculated for C_19_H_23_NI: 392.09, found: 391.95.

**Compound (II)**: The product was obtained as a white solid in 77% yield; m.p. 430–437 K. ^1^H NMR (DMSO, 300 MHz, δ, p.p.m.): 1.85 (*m*, 2H), 2.12 (*m*, 2H), 2.51 (*m*, 2H), 3.15 (*s*, 6H), 5.18 (*s*, 2H), 6.14 (*t*, *J* = 7.2 Hz, 1H), 7.11–7.51 (10H). ^13^C NMR (DMSO, 75 MHz, p.p.m.): 22.30, 25.91, 39.01, 51.19, 63.84, 126.84–141.68. ESI–MS *m*/*z* calculated for C_20_H_25_NI: 406.10, found: 406.20.

**Compound (III)**: The product was obtained as a white solid in 72% yield; m.p. 429–431 K. ^1^H NMR (DMSO, 300 MHz, δ, p.p.m.): 1.45 (*m*, 2H), 1.68 (*m*, 2H), 2.12 (*m*, 2H), 2.51 (*m*, 2H), 3.10 (*s*, 6H), 5.14 (*s*, 2H), 6.14 (*t*, *J* = 7.3 Hz, 1H), 7.06–7.51 (m, 10H) p.p.m. ^13^C NMR (DMSO, 75 MHz, p.p.m.): 25.07, 28.91, 31.91, 35.35, 54.29, 67.34, 129.89–132.50, 130.19, 142.45, 144.34, 145.02. Elemental analysis calculated for C_21_H_27_NI_*2*_: C, 46.09%; H, 4.97%; N, 2.56%; found C, 45.91%; H, 4.93%; N, 2.58%. ESI–MS *m*/*z* calculated for C_21_H_27_NI: 420.12, found: 420.20.

## Refinement   

Crystal data, data collection and structure refinement details are summarized in Table 3 ▸. For all three compounds the C-bound H atoms were included in calculated positions and treated as riding atoms: C—H = 0.93–0.99 Å with *U*_iso_(H) = 1.5*U*_eq_(C) for methyl H atoms and 1.2*U*_eq_(C) for other H atoms. Refining the structure of compound (I) in the non-centrosymmetric space group gives a value of 0.02 (3) for the Flack parameter (Flack & Bernardinelli, 1999 ▸), confirming that the direction of the polar axis has been correctly determined. The studied crystal of compound (III) was a non-merohedral twin with a ratio of two major domains of 0.374 (2):0.626 (2). The two domains are rotated from each other by 180.0° about the reciprocal axis a*, as determined by the *CELL NOW* program (Sheldrick, 2004 ▸). The final refinement was carried out using the twinned data set.

## Supplementary Material

Crystal structure: contains datablock(s) I, II, III, Global. DOI: 10.1107/S2056989015017181/su5179sup1.cif

Structure factors: contains datablock(s) I. DOI: 10.1107/S2056989015017181/su5179Isup2.hkl

Supporting information file. DOI: 10.1107/S2056989015017181/su5179Isup3.cdx

Structure factors: contains datablock(s) II. DOI: 10.1107/S2056989015017181/su5179IIsup4.hkl

Structure factors: contains datablock(s) III. DOI: 10.1107/S2056989015017181/su5179IIIsup5.hkl

Supporting information file. DOI: 10.1107/S2056989015017181/su5179Isup6.cml

Supporting information file. DOI: 10.1107/S2056989015017181/su5179IIsup7.cml

Supporting information file. DOI: 10.1107/S2056989015017181/su5179IIIsup8.cml

CCDC references: 1424320, 1424319, 1424318

Additional supporting information:  crystallographic information; 3D view; checkCIF report

## Figures and Tables

**Table 1:** Hydrogen-bond geometry (Å, °) for (I) *Cg* is the centroid of the C11–C16 ring.

*D*—H⋯*A*	*D*—H	H⋯*A*	*D*⋯*A*	*D*—H⋯*A*
C17—H17*B*⋯I2^i^	0.97	3.00	3.919 (5)	159
C7—H7⋯*Cg* ^ii^	0.93	2.84	3.030 (5)	143

Symmetry codes: (i) 
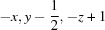
; (ii) 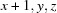.

**Table 2:** Hydrogen-bond geometry (Å, °) for (II)

*D*—H⋯*A*	*D*—H	H⋯*A*	*D*⋯*A*	*D*—H⋯*A*
C2—H2*B*⋯I2	0.97	3.06	4.001 (7)	165

**Table 3:** Experimental details

	(I)	(II)	(III)
Crystal data			
Chemical formula	C_19_H_23_IN^+^·I^−^	C_20_H_25_IN^+^·I^−^	C_21_H_27_IN^+^·I^−^
*M*_r_	519.18	533.21	547.23
Crystal system, space group	Monoclinic, *P*2_1_	Monoclinic, *C*2/*c*	Monoclinic, *P*2_1_/*c*
Temperature (K)	298	298	298
*a*, *b*, *c* (Å)	7.9254 (2), 13.6161 (3), 9.4632 (2)	37.778 (7), 6.6323 (12), 17.021 (3)	8.9423 (12), 24.058 (3), 10.3749 (13)
β (°)	103.320 (1)	100.567 (4)	103.656 (3)
*V* (Å^3^)	993.73 (4)	4192.3 (13)	2168.9 (5)
*Z*	2	8	4
Radiation type	Mo *K*α	Mo *K*α	Mo *K*α
μ (mm^−1^)	3.16	3.00	2.90
Crystal size (mm)	0.23 × 0.19 × 0.12	0.21 × 0.20 × 0.08	0.32 × 0.22 × 0.04

Data collection			
Diffractometer	Bruker SMART APEX CCD	Bruker SMART APEX CCD	Bruker SMART APEX CCD
Absorption correction	Multi-scan (*SADABS*; Bruker, 2012 ▸)	Multi-scan (*SADABS*; Bruker, 2012 ▸)	Multi-scan (*TWINABS*; Bruker, 2012 ▸)
*T*_min_, *T*_max_	0.624, 0.745	0.349, 0.745	0.273, 0.429
No. of measured, independent and observed [*I* > 2σ(*I*)] reflections	5791, 3085, 3013	16925, 3808, 3114	3961, 3961, 2941
*R*_int_	0.016	0.079	0.079
(sin θ/λ)_max_ (Å^−1^)	0.602	0.602	0.603

Refinement			
*R*[*F* ^2^ > 2σ(*F* ^2^)], *wR*(*F* ^2^), *S*	0.017, 0.038, 1.08	0.052, 0.145, 1.05	0.060, 0.138, 1.05
No. of reflections	3085	3808	3961
No. of parameters	202	210	220
No. of restraints	1	0	0
H-atom treatment	H-atom parameters constrained	H-atom parameters constrained	H-atom parameters constrained
Δρ_max_, Δρ_min_ (e Å^−3^)	0.27, −0.46	1.90, −1.98	0.82, −0.80
Absolute structure	Refined as an inversion twin	–	–
Absolute structure parameter	0.02 (3)	–	–

Computer programs: *APEX2* and *SAINT* (Bruker, 2012 ▸), *SHELXS97* (Sheldrick, 2008 ▸), *SHELXL2014* (Sheldrick, 2015 ▸), *Mercury* (Macrae *et al.*, 2008 ▸), *PLATON* (Spek, 2009 ▸) and *publCIF* (Westrip, 2010 ▸).

## supplementary crystallographic information

### (I) N-(4,4-Diphenylbut-3-en-1-yl)-N-iodomethyl-N,N-dimethylammonium iodide Crystal data

**Table:**

C_19_H_23_IN^+^·I^−^	*D*_x_ = 1.735 Mg m^−^^3^
*M**_r_* = 519.18	Melting point = 425–427 K
Monoclinic, *P*2_1_	Mo *K*α radiation, λ = 0.71073 Å
*a* = 7.9254 (2) Å	Cell parameters from 4987 reflections
*b* = 13.6161 (3) Å	θ = 2.2–25.3°
*c* = 9.4632 (2) Å	µ = 3.16 mm^−^^1^
β = 103.320 (1)°	*T* = 298 K
*V* = 993.73 (4) Å^3^	Prism, colourless
*Z* = 2	0.23 × 0.19 × 0.12 mm
*F*(000) = 500	

### (I) N-(4,4-Diphenylbut-3-en-1-yl)-N-iodomethyl-N,N-dimethylammonium iodide Data collection

**Table:**

Bruker SMART APEX CCD diffractometer	3085 independent reflections
Radiation source: fine-focus sealed tube	3013 reflections with *I* > 2σ(*I*)
Graphite monochromator	*R*_int_ = 0.016
Detector resolution: 8.333 pixels mm^-1^	θ_max_ = 25.3°, θ_min_ = 2.2°
ω–scans	*h* = −9→9
Absorption correction: multi-scan (*SADABS*; Bruker, 2012)	*k* = −16→16
*T*_min_ = 0.624, *T*_max_ = 0.745	*l* = −11→11
5791 measured reflections	

### (I) N-(4,4-Diphenylbut-3-en-1-yl)-N-iodomethyl-N,N-dimethylammonium iodide Refinement

**Table:**

Refinement on *F*^2^	Secondary atom site location: difference Fourier map
Least-squares matrix: full	Hydrogen site location: inferred from neighbouring sites
*R*[*F*^2^ > 2σ(*F*^2^)] = 0.017	H-atom parameters constrained
*wR*(*F*^2^) = 0.038	*w* = 1/[σ^2^(*F*_o_^2^) + (0.0138*P*)^2^ + 0.0203*P*] where *P* = (*F*_o_^2^ + 2*F*_c_^2^)/3
*S* = 1.08	(Δ/σ)_max_ = 0.001
3085 reflections	Δρ_max_ = 0.27 e Å^−^^3^
202 parameters	Δρ_min_ = −0.46 e Å^−^^3^
1 restraint	Absolute structure: Refined as an inversion twin
Primary atom site location: structure-invariant direct methods	Absolute structure parameter: 0.02 (3)

### (I) N-(4,4-Diphenylbut-3-en-1-yl)-N-iodomethyl-N,N-dimethylammonium iodide Special details

**Table:**

Geometry. All e.s.d.'s (except the e.s.d. in the dihedral angle between two l.s. planes) are estimated using the full covariance matrix. The cell e.s.d.'s are taken into account individually in the estimation of e.s.d.'s in distances, angles and torsion angles; correlations between e.s.d.'s in cell parameters are only used when they are defined by crystal symmetry. An approximate (isotropic) treatment of cell e.s.d.'s is used for estimating e.s.d.'s involving l.s. planes.
Refinement. Refined as a 2-component inversion twin.

### (I) N-(4,4-Diphenylbut-3-en-1-yl)-N-iodomethyl-N,N-dimethylammonium iodide Fractional atomic coordinates and isotropic or equivalent isotropic displacement parameters (Å^2^)

**Table:**

	*x*	*y*	*z*	*U*_iso_*/*U*_eq_	
I1	−0.65685 (3)	0.06224 (2)	0.13054 (3)	0.04839 (9)	
I2	0.06519 (4)	0.44189 (2)	0.91487 (3)	0.04368 (9)	
N1	−0.3770 (4)	0.2023 (3)	0.0555 (4)	0.0376 (8)	
C1	−0.1849 (5)	0.2322 (4)	0.0948 (5)	0.0402 (10)	
H1A	−0.1144	0.1733	0.1019	0.048*	
H1B	−0.1609	0.2719	0.0166	0.048*	
C2	−0.1311 (5)	0.2891 (3)	0.2358 (5)	0.0409 (11)	
H2A	−0.1888	0.3524	0.2249	0.049*	
H2B	−0.1676	0.2536	0.3124	0.049*	
C3	0.0613 (6)	0.3044 (3)	0.2779 (5)	0.0382 (10)	
H3	0.1101	0.3408	0.2143	0.046*	
C4	0.1686 (5)	0.2711 (3)	0.3970 (4)	0.0329 (9)	
C5	0.3545 (5)	0.3021 (3)	0.4380 (4)	0.0349 (9)	
C6	0.4815 (6)	0.2388 (4)	0.5116 (5)	0.0457 (11)	
H6	0.4514	0.1752	0.5318	0.055*	
C7	0.6528 (6)	0.2687 (4)	0.5557 (5)	0.0546 (13)	
H7	0.7362	0.2248	0.6040	0.065*	
C8	0.7001 (6)	0.3612 (5)	0.5292 (6)	0.0598 (15)	
H8	0.8148	0.3812	0.5610	0.072*	
C9	0.5768 (7)	0.4253 (4)	0.4549 (7)	0.0661 (16)	
H9	0.6087	0.4887	0.4357	0.079*	
C10	0.4056 (6)	0.3959 (4)	0.4085 (6)	0.0532 (13)	
H10	0.3239	0.4395	0.3569	0.064*	
C11	0.1128 (5)	0.2030 (3)	0.5013 (4)	0.0326 (9)	
C12	0.0439 (6)	0.1106 (3)	0.4575 (5)	0.0422 (10)	
H12	0.0314	0.0909	0.3616	0.051*	
C13	−0.0060 (6)	0.0480 (3)	0.5561 (5)	0.0500 (12)	
H13	−0.0522	−0.0134	0.5258	0.060*	
C14	0.0124 (6)	0.0761 (4)	0.6978 (5)	0.0523 (13)	
H14	−0.0198	0.0334	0.7637	0.063*	
C15	0.0784 (6)	0.1674 (4)	0.7431 (5)	0.0467 (12)	
H15	0.0883	0.1871	0.8388	0.056*	
C16	0.1298 (5)	0.2295 (4)	0.6457 (5)	0.0403 (10)	
H16	0.1768	0.2905	0.6774	0.048*	
C17	−0.4036 (5)	0.1302 (3)	0.1685 (5)	0.0411 (10)	
H17A	−0.3821	0.1637	0.2614	0.049*	
H17B	−0.3174	0.0787	0.1761	0.049*	
C18	−0.4928 (7)	0.2892 (4)	0.0510 (7)	0.0633 (15)	
H18A	−0.6106	0.2703	0.0100	0.095*	
H18B	−0.4590	0.3397	−0.0076	0.095*	
H18C	−0.4832	0.3135	0.1478	0.095*	
C19	−0.4089 (7)	0.1546 (5)	−0.0910 (5)	0.0596 (14)	
H19A	−0.5290	0.1369	−0.1218	0.089*	
H19B	−0.3385	0.0967	−0.0855	0.089*	
H19C	−0.3796	0.1997	−0.1596	0.089*	

### (I) N-(4,4-Diphenylbut-3-en-1-yl)-N-iodomethyl-N,N-dimethylammonium iodide Atomic displacement parameters (Å^2^)

**Table:**

	*U*^11^	*U*^22^	*U*^33^	*U*^12^	*U*^13^	*U*^23^
I1	0.03965 (16)	0.05161 (19)	0.05521 (18)	−0.00709 (15)	0.01359 (13)	−0.00180 (15)
I2	0.04705 (17)	0.03979 (15)	0.04515 (16)	0.00372 (15)	0.01257 (12)	0.00767 (14)
N1	0.0317 (17)	0.043 (2)	0.0351 (19)	−0.0020 (17)	0.0010 (15)	0.0047 (16)
C1	0.029 (2)	0.051 (3)	0.039 (2)	−0.005 (2)	0.0042 (18)	0.006 (2)
C2	0.035 (2)	0.043 (3)	0.042 (3)	0.001 (2)	0.004 (2)	−0.001 (2)
C3	0.038 (2)	0.037 (2)	0.038 (2)	−0.004 (2)	0.0058 (19)	0.003 (2)
C4	0.034 (2)	0.031 (2)	0.033 (2)	−0.0022 (18)	0.0073 (18)	−0.0027 (18)
C5	0.033 (2)	0.037 (2)	0.035 (2)	−0.002 (2)	0.0087 (18)	−0.0047 (19)
C6	0.037 (3)	0.055 (3)	0.046 (3)	0.003 (2)	0.012 (2)	0.011 (2)
C7	0.033 (2)	0.083 (4)	0.049 (3)	0.007 (3)	0.010 (2)	0.009 (3)
C8	0.034 (3)	0.080 (4)	0.065 (4)	−0.009 (3)	0.011 (3)	−0.014 (3)
C9	0.049 (3)	0.048 (4)	0.103 (4)	−0.019 (3)	0.021 (3)	−0.017 (3)
C10	0.040 (3)	0.037 (2)	0.082 (4)	−0.003 (2)	0.012 (3)	0.003 (3)
C11	0.0266 (19)	0.035 (2)	0.035 (2)	0.0031 (18)	0.0054 (17)	0.0010 (18)
C12	0.043 (2)	0.040 (2)	0.045 (2)	−0.002 (2)	0.013 (2)	−0.004 (2)
C13	0.054 (3)	0.032 (3)	0.068 (3)	−0.001 (2)	0.022 (2)	0.004 (2)
C14	0.046 (3)	0.054 (3)	0.061 (3)	0.009 (3)	0.019 (2)	0.023 (3)
C15	0.043 (3)	0.065 (3)	0.031 (2)	0.007 (3)	0.008 (2)	0.007 (2)
C16	0.031 (2)	0.048 (3)	0.040 (2)	−0.004 (2)	0.0043 (19)	−0.004 (2)
C17	0.035 (2)	0.049 (3)	0.037 (2)	−0.005 (2)	0.0035 (18)	0.005 (2)
C18	0.041 (3)	0.049 (3)	0.091 (4)	0.009 (3)	−0.002 (3)	0.013 (3)
C19	0.055 (3)	0.084 (4)	0.036 (3)	−0.014 (3)	0.003 (2)	0.001 (3)

### (I) N-(4,4-Diphenylbut-3-en-1-yl)-N-iodomethyl-N,N-dimethylammonium iodide Geometric parameters (Å, º)

**Table:**

I1—C17	2.164 (4)	C8—H8	0.9300
I1—I2^i^	3.5640 (4)	C9—C10	1.386 (7)
N1—C18	1.492 (6)	C9—H9	0.9300
N1—C19	1.499 (6)	C10—H10	0.9300
N1—C17	1.502 (5)	C11—C16	1.390 (6)
N1—C1	1.536 (5)	C11—C12	1.396 (6)
C1—C2	1.517 (6)	C12—C13	1.387 (6)
C1—H1A	0.9700	C12—H12	0.9300
C1—H1B	0.9700	C13—C14	1.370 (7)
C2—C3	1.499 (6)	C13—H13	0.9300
C2—H2A	0.9700	C14—C15	1.379 (7)
C2—H2B	0.9700	C14—H14	0.9300
C3—C4	1.326 (6)	C15—C16	1.379 (6)
C3—H3	0.9300	C15—H15	0.9300
C4—C11	1.493 (6)	C16—H16	0.9300
C4—C5	1.495 (6)	C17—H17A	0.9700
C5—C6	1.385 (6)	C17—H17B	0.9700
C5—C10	1.388 (6)	C18—H18A	0.9600
C6—C7	1.387 (7)	C18—H18B	0.9600
C6—H6	0.9300	C18—H18C	0.9600
C7—C8	1.353 (8)	C19—H19A	0.9600
C7—H7	0.9300	C19—H19B	0.9600
C8—C9	1.376 (8)	C19—H19C	0.9600
			
C17—I1—I2^i^	176.81 (12)	C9—C10—C5	120.8 (5)
C18—N1—C19	110.2 (4)	C9—C10—H10	119.6
C18—N1—C17	110.7 (4)	C5—C10—H10	119.6
C19—N1—C17	110.7 (4)	C16—C11—C12	118.0 (4)
C18—N1—C1	111.4 (4)	C16—C11—C4	120.8 (4)
C19—N1—C1	106.6 (3)	C12—C11—C4	121.2 (4)
C17—N1—C1	107.1 (3)	C13—C12—C11	120.5 (4)
C2—C1—N1	114.2 (3)	C13—C12—H12	119.8
C2—C1—H1A	108.7	C11—C12—H12	119.8
N1—C1—H1A	108.7	C14—C13—C12	120.3 (5)
C2—C1—H1B	108.7	C14—C13—H13	119.9
N1—C1—H1B	108.7	C12—C13—H13	119.9
H1A—C1—H1B	107.6	C13—C14—C15	120.2 (4)
C3—C2—C1	111.6 (4)	C13—C14—H14	119.9
C3—C2—H2A	109.3	C15—C14—H14	119.9
C1—C2—H2A	109.3	C14—C15—C16	119.7 (4)
C3—C2—H2B	109.3	C14—C15—H15	120.2
C1—C2—H2B	109.3	C16—C15—H15	120.2
H2A—C2—H2B	108.0	C15—C16—C11	121.4 (4)
C4—C3—C2	126.3 (4)	C15—C16—H16	119.3
C4—C3—H3	116.8	C11—C16—H16	119.3
C2—C3—H3	116.8	N1—C17—I1	116.0 (3)
C3—C4—C11	123.0 (4)	N1—C17—H17A	108.3
C3—C4—C5	121.6 (4)	I1—C17—H17A	108.3
C11—C4—C5	115.3 (3)	N1—C17—H17B	108.3
C6—C5—C10	117.5 (4)	I1—C17—H17B	108.3
C6—C5—C4	120.9 (4)	H17A—C17—H17B	107.4
C10—C5—C4	121.6 (4)	N1—C18—H18A	109.5
C5—C6—C7	121.1 (5)	N1—C18—H18B	109.5
C5—C6—H6	119.4	H18A—C18—H18B	109.5
C7—C6—H6	119.4	N1—C18—H18C	109.5
C8—C7—C6	120.7 (5)	H18A—C18—H18C	109.5
C8—C7—H7	119.6	H18B—C18—H18C	109.5
C6—C7—H7	119.6	N1—C19—H19A	109.5
C7—C8—C9	119.4 (5)	N1—C19—H19B	109.5
C7—C8—H8	120.3	H19A—C19—H19B	109.5
C9—C8—H8	120.3	N1—C19—H19C	109.5
C8—C9—C10	120.5 (5)	H19A—C19—H19C	109.5
C8—C9—H9	119.8	H19B—C19—H19C	109.5
C10—C9—H9	119.8		
			
C18—N1—C1—C2	55.2 (5)	C6—C5—C10—C9	−1.8 (8)
C19—N1—C1—C2	175.5 (4)	C4—C5—C10—C9	176.1 (5)
C17—N1—C1—C2	−65.9 (5)	C3—C4—C11—C16	−120.5 (5)
N1—C1—C2—C3	172.5 (4)	C5—C4—C11—C16	57.8 (5)
C1—C2—C3—C4	−117.9 (5)	C3—C4—C11—C12	60.2 (6)
C2—C3—C4—C11	6.6 (7)	C5—C4—C11—C12	−121.5 (4)
C2—C3—C4—C5	−171.6 (4)	C16—C11—C12—C13	0.2 (6)
C3—C4—C5—C6	−148.1 (5)	C4—C11—C12—C13	179.5 (4)
C11—C4—C5—C6	33.5 (5)	C11—C12—C13—C14	−0.3 (7)
C3—C4—C5—C10	34.0 (6)	C12—C13—C14—C15	0.9 (7)
C11—C4—C5—C10	−144.3 (4)	C13—C14—C15—C16	−1.5 (7)
C10—C5—C6—C7	0.9 (7)	C14—C15—C16—C11	1.5 (7)
C4—C5—C6—C7	−177.0 (4)	C12—C11—C16—C15	−0.8 (6)
C5—C6—C7—C8	0.7 (7)	C4—C11—C16—C15	179.9 (4)
C6—C7—C8—C9	−1.4 (8)	C18—N1—C17—I1	64.6 (4)
C7—C8—C9—C10	0.5 (9)	C19—N1—C17—I1	−57.9 (4)
C8—C9—C10—C5	1.1 (9)	C1—N1—C17—I1	−173.8 (3)

Symmetry code: (i) −*x*−1, *y*−1/2, −*z*+1.

### (I) N-(4,4-Diphenylbut-3-en-1-yl)-N-iodomethyl-N,N-dimethylammonium iodide Hydrogen-bond geometry (Å, º)

Cg is the centroid of the C11–C16 ring.

**Table:**

*D*—H···*A*	*D*—H	H···*A*	*D*···*A*	*D*—H···*A*
C17—H17*B*···I2^ii^	0.97	3.00	3.919 (5)	159
C7—H7···*Cg*^iii^	0.93	2.84	3.030 (5)	143

Symmetry codes: (ii) −*x*, *y*−1/2, −*z*+1; (iii) *x*+1, *y*, *z*.

### (II) N-(5,5-Diphenylpent-4-en-1-yl)-N-iodomethyl-N,N-dimethylammonium iodide Crystal data

**Table:**

C_20_H_25_IN^+^·I^−^	*D*_x_ = 1.690 Mg m^−^^3^
*M**_r_* = 533.21	Melting point = 430–431 K
Monoclinic, *C*2/*c*	Mo *K*α radiation, λ = 0.71073 Å
*a* = 37.778 (7) Å	Cell parameters from 4726 reflections
*b* = 6.6323 (12) Å	θ = 2.2–25.3°
*c* = 17.021 (3) Å	µ = 3.00 mm^−^^1^
β = 100.567 (4)°	*T* = 298 K
*V* = 4192.3 (13) Å^3^	Platy-prism, colourless
*Z* = 8	0.21 × 0.20 × 0.08 mm
*F*(000) = 2064	

### (II) N-(5,5-Diphenylpent-4-en-1-yl)-N-iodomethyl-N,N-dimethylammonium iodide Data collection

**Table:**

Bruker SMART APEX CCD diffractometer	3808 independent reflections
Radiation source: fine-focus sealed tube	3114 reflections with *I* > 2σ(*I*)
Graphite monochromator	*R*_int_ = 0.079
ω–scans	θ_max_ = 25.4°, θ_min_ = 2.2°
Absorption correction: multi-scan (*SADABS*; Bruker, 2012)	*h* = −45→45
*T*_min_ = 0.349, *T*_max_ = 0.745	*k* = −7→7
16925 measured reflections	*l* = −20→20

### (II) N-(5,5-Diphenylpent-4-en-1-yl)-N-iodomethyl-N,N-dimethylammonium iodide Refinement

**Table:**

Refinement on *F*^2^	Primary atom site location: structure-invariant direct methods
Least-squares matrix: full	Secondary atom site location: difference Fourier map
*R*[*F*^2^ > 2σ(*F*^2^)] = 0.052	Hydrogen site location: inferred from neighbouring sites
*wR*(*F*^2^) = 0.145	H-atom parameters constrained
*S* = 1.05	*w* = 1/[σ^2^(*F*_o_^2^) + (0.0918*P*)^2^] where *P* = (*F*_o_^2^ + 2*F*_c_^2^)/3
3808 reflections	(Δ/σ)_max_ = 0.001
210 parameters	Δρ_max_ = 1.90 e Å^−^^3^
0 restraints	Δρ_min_ = −1.98 e Å^−^^3^

### (II) N-(5,5-Diphenylpent-4-en-1-yl)-N-iodomethyl-N,N-dimethylammonium iodide Special details

**Table:**

Geometry. All e.s.d.'s (except the e.s.d. in the dihedral angle between two l.s. planes) are estimated using the full covariance matrix. The cell e.s.d.'s are taken into account individually in the estimation of e.s.d.'s in distances, angles and torsion angles; correlations between e.s.d.'s in cell parameters are only used when they are defined by crystal symmetry. An approximate (isotropic) treatment of cell e.s.d.'s is used for estimating e.s.d.'s involving l.s. planes.

### (II) N-(5,5-Diphenylpent-4-en-1-yl)-N-iodomethyl-N,N-dimethylammonium iodide Fractional atomic coordinates and isotropic or equivalent isotropic displacement parameters (Å^2^)

**Table:**

	*x*	*y*	*z*	*U*_iso_*/*U*_eq_	
I1	0.25244 (2)	−0.01229 (6)	0.13640 (2)	0.04488 (19)	
I2	0.30512 (2)	0.07869 (7)	0.42246 (3)	0.04999 (19)	
N1	0.31227 (12)	0.3200 (8)	0.1496 (2)	0.0383 (11)	
C1	0.32956 (18)	0.4989 (9)	0.1981 (4)	0.0446 (15)	
H1A	0.3463	0.5629	0.1690	0.053*	
H1B	0.3109	0.5965	0.2029	0.053*	
C2	0.34941 (19)	0.4459 (10)	0.2810 (4)	0.0503 (16)	
H2A	0.3713	0.3736	0.2770	0.060*	
H2B	0.3345	0.3583	0.3069	0.060*	
C3	0.35877 (18)	0.6363 (11)	0.3312 (4)	0.0498 (15)	
H3A	0.3769	0.7134	0.3107	0.060*	
H3B	0.3375	0.7199	0.3284	0.060*	
C4	0.37268 (18)	0.5764 (12)	0.4160 (4)	0.0536 (17)	
H4	0.3577	0.4935	0.4394	0.064*	
C5	0.40427 (16)	0.6276 (11)	0.4628 (3)	0.0476 (15)	
C6	0.41456 (18)	0.5452 (12)	0.5446 (4)	0.0537 (18)	
C7	0.4042 (2)	0.3523 (14)	0.5653 (4)	0.066 (2)	
H7	0.3905	0.2719	0.5265	0.080*	
C8	0.4140 (2)	0.2791 (17)	0.6421 (5)	0.083 (3)	
H8	0.4068	0.1506	0.6544	0.099*	
C9	0.4346 (2)	0.396 (2)	0.7012 (5)	0.095 (4)	
H9	0.4411	0.3468	0.7530	0.114*	
C10	0.4453 (2)	0.586 (2)	0.6822 (5)	0.098 (4)	
H10	0.4592	0.6652	0.7213	0.117*	
C11	0.43528 (19)	0.6603 (16)	0.6054 (4)	0.073 (2)	
H11	0.4425	0.7893	0.5937	0.088*	
C12	0.42943 (16)	0.7710 (12)	0.4341 (3)	0.0494 (16)	
C13	0.4190 (2)	0.9644 (13)	0.4097 (5)	0.067 (2)	
H13	0.3957	1.0067	0.4121	0.080*	
C14	0.4417 (3)	1.0942 (16)	0.3824 (6)	0.087 (3)	
H14	0.4339	1.2226	0.3655	0.104*	
C15	0.4761 (3)	1.036 (2)	0.3796 (7)	0.097 (4)	
H15	0.4914	1.1257	0.3604	0.117*	
C16	0.4882 (2)	0.849 (2)	0.4045 (5)	0.091 (3)	
H16	0.5118	0.8115	0.4026	0.109*	
C17	0.46478 (18)	0.7137 (15)	0.4330 (4)	0.067 (2)	
H17	0.4729	0.5865	0.4509	0.080*	
C18	0.28176 (16)	0.2484 (10)	0.1869 (3)	0.0425 (14)	
H18A	0.2648	0.3587	0.1853	0.051*	
H18B	0.2911	0.2201	0.2428	0.051*	
C19	0.33945 (17)	0.1549 (11)	0.1464 (4)	0.0509 (16)	
H19A	0.3288	0.0516	0.1101	0.076*	
H19B	0.3468	0.0982	0.1988	0.076*	
H19C	0.3601	0.2098	0.1283	0.076*	
C20	0.29821 (18)	0.3908 (10)	0.0658 (3)	0.0495 (16)	
H20A	0.2870	0.2801	0.0343	0.074*	
H20B	0.3178	0.4416	0.0427	0.074*	
H20C	0.2808	0.4959	0.0668	0.074*	

### (II) N-(5,5-Diphenylpent-4-en-1-yl)-N-iodomethyl-N,N-dimethylammonium iodide Atomic displacement parameters (Å^2^)

**Table:**

	*U*^11^	*U*^22^	*U*^33^	*U*^12^	*U*^13^	*U*^23^
I1	0.0501 (3)	0.0520 (3)	0.0289 (3)	−0.01023 (17)	−0.00240 (18)	0.00087 (16)
I2	0.0512 (3)	0.0512 (3)	0.0461 (3)	0.00415 (19)	0.0052 (2)	0.01397 (19)
N1	0.041 (3)	0.050 (3)	0.021 (2)	−0.003 (2)	−0.0006 (19)	−0.002 (2)
C1	0.052 (4)	0.047 (4)	0.033 (3)	−0.013 (3)	0.001 (3)	−0.003 (3)
C2	0.052 (4)	0.062 (4)	0.031 (3)	−0.008 (3)	−0.009 (3)	−0.005 (3)
C3	0.044 (3)	0.062 (4)	0.039 (3)	−0.010 (3)	−0.003 (3)	−0.011 (3)
C4	0.047 (4)	0.082 (5)	0.028 (3)	−0.012 (3)	−0.001 (3)	−0.011 (3)
C5	0.042 (3)	0.067 (4)	0.031 (3)	−0.009 (3)	0.000 (2)	−0.019 (3)
C6	0.035 (3)	0.089 (5)	0.036 (3)	0.001 (3)	0.004 (3)	−0.008 (3)
C7	0.064 (5)	0.088 (6)	0.046 (4)	0.000 (4)	0.008 (3)	−0.002 (4)
C8	0.066 (5)	0.126 (8)	0.058 (5)	0.013 (5)	0.018 (4)	0.021 (5)
C9	0.051 (5)	0.188 (12)	0.044 (5)	0.007 (6)	0.002 (4)	0.014 (6)
C10	0.059 (5)	0.190 (12)	0.039 (4)	−0.033 (7)	−0.004 (4)	−0.024 (6)
C11	0.057 (4)	0.118 (7)	0.042 (4)	−0.023 (5)	0.001 (3)	−0.021 (4)
C12	0.040 (3)	0.075 (5)	0.029 (3)	−0.008 (3)	−0.003 (2)	−0.016 (3)
C13	0.057 (5)	0.075 (5)	0.061 (5)	−0.012 (4)	−0.007 (4)	−0.020 (4)
C14	0.079 (6)	0.092 (7)	0.079 (6)	−0.027 (5)	−0.014 (5)	0.009 (5)
C15	0.073 (7)	0.136 (10)	0.076 (6)	−0.044 (6)	−0.004 (5)	0.025 (7)
C16	0.047 (4)	0.167 (11)	0.056 (5)	−0.019 (6)	0.004 (4)	−0.005 (6)
C17	0.043 (4)	0.111 (7)	0.042 (4)	−0.004 (4)	−0.002 (3)	−0.004 (4)
C18	0.044 (3)	0.058 (4)	0.025 (3)	−0.009 (3)	0.003 (2)	−0.004 (3)
C19	0.048 (4)	0.058 (4)	0.044 (3)	0.005 (3)	0.002 (3)	−0.011 (3)
C20	0.057 (4)	0.061 (4)	0.028 (3)	−0.004 (3)	0.000 (3)	0.006 (3)

### (II) N-(5,5-Diphenylpent-4-en-1-yl)-N-iodomethyl-N,N-dimethylammonium iodide Geometric parameters (Å, º)

**Table:**

I1—C18	2.146 (6)	C9—C10	1.381 (16)
I1—I2^i^	3.5058 (7)	C9—H9	0.9300
N1—C18	1.492 (7)	C10—C11	1.382 (12)
N1—C20	1.504 (7)	C10—H10	0.9300
N1—C19	1.509 (8)	C11—H11	0.9300
N1—C1	1.522 (8)	C12—C13	1.383 (11)
C1—C2	1.513 (9)	C12—C17	1.392 (9)
C1—H1A	0.9700	C13—C14	1.357 (12)
C1—H1B	0.9700	C13—H13	0.9300
C2—C3	1.530 (9)	C14—C15	1.361 (14)
C2—H2A	0.9700	C14—H14	0.9300
C2—H2B	0.9700	C15—C16	1.363 (16)
C3—C4	1.496 (9)	C15—H15	0.9300
C3—H3A	0.9700	C16—C17	1.410 (13)
C3—H3B	0.9700	C16—H16	0.9300
C4—C5	1.351 (9)	C17—H17	0.9300
C4—H4	0.9300	C18—H18A	0.9700
C5—C6	1.480 (10)	C18—H18B	0.9700
C5—C12	1.489 (9)	C19—H19A	0.9600
C6—C7	1.402 (12)	C19—H19B	0.9600
C6—C11	1.404 (10)	C19—H19C	0.9600
C7—C8	1.379 (11)	C20—H20A	0.9600
C7—H7	0.9300	C20—H20B	0.9600
C8—C9	1.390 (14)	C20—H20C	0.9600
C8—H8	0.9300		
			
C18—I1—I2^i^	170.16 (15)	C9—C10—C11	120.3 (9)
C18—N1—C20	109.7 (4)	C9—C10—H10	119.9
C18—N1—C19	111.6 (5)	C11—C10—H10	119.9
C20—N1—C19	108.5 (5)	C10—C11—C6	121.6 (9)
C18—N1—C1	107.8 (4)	C10—C11—H11	119.2
C20—N1—C1	108.2 (5)	C6—C11—H11	119.2
C19—N1—C1	111.0 (5)	C13—C12—C17	118.1 (7)
C2—C1—N1	114.4 (5)	C13—C12—C5	121.8 (6)
C2—C1—H1A	108.6	C17—C12—C5	120.1 (7)
N1—C1—H1A	108.6	C14—C13—C12	121.8 (8)
C2—C1—H1B	108.6	C14—C13—H13	119.1
N1—C1—H1B	108.6	C12—C13—H13	119.1
H1A—C1—H1B	107.6	C13—C14—C15	120.0 (10)
C1—C2—C3	110.7 (6)	C13—C14—H14	120.0
C1—C2—H2A	109.5	C15—C14—H14	120.0
C3—C2—H2A	109.5	C14—C15—C16	121.0 (9)
C1—C2—H2B	109.5	C14—C15—H15	119.5
C3—C2—H2B	109.5	C16—C15—H15	119.5
H2A—C2—H2B	108.1	C15—C16—C17	119.3 (9)
C4—C3—C2	108.9 (6)	C15—C16—H16	120.3
C4—C3—H3A	109.9	C17—C16—H16	120.3
C2—C3—H3A	109.9	C12—C17—C16	119.7 (9)
C4—C3—H3B	109.9	C12—C17—H17	120.1
C2—C3—H3B	109.9	C16—C17—H17	120.1
H3A—C3—H3B	108.3	N1—C18—I1	117.9 (4)
C5—C4—C3	128.2 (7)	N1—C18—H18A	107.8
C5—C4—H4	115.9	I1—C18—H18A	107.8
C3—C4—H4	115.9	N1—C18—H18B	107.8
C4—C5—C6	120.9 (6)	I1—C18—H18B	107.8
C4—C5—C12	121.0 (6)	H18A—C18—H18B	107.2
C6—C5—C12	118.1 (5)	N1—C19—H19A	109.5
C7—C6—C11	117.0 (7)	N1—C19—H19B	109.5
C7—C6—C5	122.5 (6)	H19A—C19—H19B	109.5
C11—C6—C5	120.5 (7)	N1—C19—H19C	109.5
C8—C7—C6	121.4 (8)	H19A—C19—H19C	109.5
C8—C7—H7	119.3	H19B—C19—H19C	109.5
C6—C7—H7	119.3	N1—C20—H20A	109.5
C7—C8—C9	120.4 (10)	N1—C20—H20B	109.5
C7—C8—H8	119.8	H20A—C20—H20B	109.5
C9—C8—H8	119.8	N1—C20—H20C	109.5
C10—C9—C8	119.3 (8)	H20A—C20—H20C	109.5
C10—C9—H9	120.3	H20B—C20—H20C	109.5
C8—C9—H9	120.3		
			
C18—N1—C1—C2	69.2 (7)	C7—C6—C11—C10	0.4 (12)
C20—N1—C1—C2	−172.2 (5)	C5—C6—C11—C10	−179.9 (8)
C19—N1—C1—C2	−53.3 (7)	C4—C5—C12—C13	57.8 (9)
N1—C1—C2—C3	−167.8 (5)	C6—C5—C12—C13	−121.2 (7)
C1—C2—C3—C4	170.6 (6)	C4—C5—C12—C17	−123.6 (8)
C2—C3—C4—C5	124.9 (8)	C6—C5—C12—C17	57.4 (8)
C3—C4—C5—C6	−176.7 (7)	C17—C12—C13—C14	2.5 (11)
C3—C4—C5—C12	4.3 (12)	C5—C12—C13—C14	−179.0 (7)
C4—C5—C6—C7	32.1 (11)	C12—C13—C14—C15	−1.0 (14)
C12—C5—C6—C7	−148.9 (7)	C13—C14—C15—C16	−0.4 (16)
C4—C5—C6—C11	−147.5 (7)	C14—C15—C16—C17	0.4 (16)
C12—C5—C6—C11	31.5 (10)	C13—C12—C17—C16	−2.5 (10)
C11—C6—C7—C8	0.0 (11)	C5—C12—C17—C16	179.0 (6)
C5—C6—C7—C8	−179.7 (7)	C15—C16—C17—C12	1.1 (13)
C6—C7—C8—C9	0.0 (12)	C20—N1—C18—I1	64.9 (6)
C7—C8—C9—C10	−0.2 (14)	C19—N1—C18—I1	−55.4 (5)
C8—C9—C10—C11	0.6 (15)	C1—N1—C18—I1	−177.5 (4)
C9—C10—C11—C6	−0.7 (14)		

Symmetry code: (i) −*x*+1/2, *y*−1/2, −*z*+1/2.

### (II) N-(5,5-Diphenylpent-4-en-1-yl)-N-iodomethyl-N,N-dimethylammonium iodide Hydrogen-bond geometry (Å, º)

**Table:**

*D*—H···*A*	*D*—H	H···*A*	*D*···*A*	*D*—H···*A*
C2—H2*B*···I2	0.97	3.06	4.001 (7)	165

### (III) N-(6,6-Diphenylhex-5-en-1-yl)-N-iodomethyl-N,N-dimethylammonium iodide Crystal data

**Table:**

C_21_H_27_IN^+^·I^−^	*D*_x_ = 1.676 Mg m^−^^3^
*M**_r_* = 547.23	Melting point = 429–431 K
Monoclinic, *P*2_1_/*c*	Mo *K*α radiation, λ = 0.71073 Å
*a* = 8.9423 (12) Å	Cell parameters from 3046 reflections
*b* = 24.058 (3) Å	θ = 2.3–24.4°
*c* = 10.3749 (13) Å	µ = 2.90 mm^−^^1^
β = 103.656 (3)°	*T* = 298 K
*V* = 2168.9 (5) Å^3^	Prism, colourless
*Z* = 4	0.32 × 0.22 × 0.04 mm
*F*(000) = 1064	

### (III) N-(6,6-Diphenylhex-5-en-1-yl)-N-iodomethyl-N,N-dimethylammonium iodide Data collection

**Table:**

Bruker SMART APEX CCD diffractometer	3961 independent reflections
Radiation source: fine-focus sealed tube	2941 reflections with *I* > 2σ(*I*)
Graphite monochromator	*R*_int_ = 0.079
Detector resolution: 8.333 pixels mm^-1^	θ_max_ = 25.4°, θ_min_ = 1.7°
ω–scans	*h* = −10→10
Absorption correction: multi-scan (*TWINABS*; Bruker, 2012)	*k* = 0→28
*T*_min_ = 0.273, *T*_max_ = 0.429	*l* = 0→12
3961 measured reflections	

### (III) N-(6,6-Diphenylhex-5-en-1-yl)-N-iodomethyl-N,N-dimethylammonium iodide Refinement

**Table:**

Refinement on *F*^2^	Primary atom site location: structure-invariant direct methods
Least-squares matrix: full	Secondary atom site location: difference Fourier map
*R*[*F*^2^ > 2σ(*F*^2^)] = 0.060	Hydrogen site location: inferred from neighbouring sites
*wR*(*F*^2^) = 0.138	H-atom parameters constrained
*S* = 1.05	*w* = 1/[σ^2^(*F*_o_^2^) + (0.0515*P*)^2^ + 2.9321*P*] where *P* = (*F*_o_^2^ + 2*F*_c_^2^)/3
3961 reflections	(Δ/σ)_max_ < 0.001
220 parameters	Δρ_max_ = 0.82 e Å^−^^3^
0 restraints	Δρ_min_ = −0.80 e Å^−^^3^

### (III) N-(6,6-Diphenylhex-5-en-1-yl)-N-iodomethyl-N,N-dimethylammonium iodide Special details

**Table:**

Geometry. All e.s.d.'s (except the e.s.d. in the dihedral angle between two l.s. planes) are estimated using the full covariance matrix. The cell e.s.d.'s are taken into account individually in the estimation of e.s.d.'s in distances, angles and torsion angles; correlations between e.s.d.'s in cell parameters are only used when they are defined by crystal symmetry. An approximate (isotropic) treatment of cell e.s.d.'s is used for estimating e.s.d.'s involving l.s. planes.
Refinement. Refined as a 2-component twin. The studied crystal was a nonmerohedral twin with a ratio of two major domains of 0.374 (2):0.626 (2). The two domains were rotated from each other by 180.0° about the recipocal axis (1 0 0), which was determined by the CELL NOW program (Sheldrick, 2004). The final refinement was carried out using twinned data set.

### (III) N-(6,6-Diphenylhex-5-en-1-yl)-N-iodomethyl-N,N-dimethylammonium iodide Fractional atomic coordinates and isotropic or equivalent isotropic displacement parameters (Å^2^)

**Table:**

	*x*	*y*	*z*	*U*_iso_*/*U*_eq_	
I1	−0.29957 (8)	0.44339 (3)	0.01740 (6)	0.0597 (2)	
I2	0.17494 (8)	0.51476 (3)	0.26901 (6)	0.0598 (2)	
N1	−0.3411 (8)	0.4415 (4)	0.3070 (7)	0.056 (2)	
C1	−0.1733 (11)	0.4319 (4)	0.3631 (10)	0.063 (3)	
H1A	−0.1159	0.4525	0.3103	0.076*	
H1B	−0.1459	0.4469	0.4524	0.076*	
C2	−0.1252 (12)	0.3731 (4)	0.3678 (12)	0.072 (3)	
H2A	−0.1407	0.3584	0.2786	0.087*	
H2B	−0.1860	0.3512	0.4153	0.087*	
C3	0.0467 (11)	0.3699 (4)	0.4394 (11)	0.068 (3)	
H3A	0.1029	0.3971	0.4001	0.082*	
H3B	0.0578	0.3800	0.5317	0.082*	
C4	0.1182 (12)	0.3134 (4)	0.4333 (15)	0.091 (4)	
H4A	0.1013	0.3019	0.3413	0.109*	
H4B	0.0681	0.2866	0.4789	0.109*	
C5	0.2892 (10)	0.3135 (4)	0.4957 (12)	0.068 (3)	
H5	0.3381	0.3478	0.5120	0.082*	
C6	0.3743 (10)	0.2685 (3)	0.5287 (10)	0.055 (2)	
C7	0.5451 (11)	0.2703 (3)	0.5813 (9)	0.052 (2)	
C8	0.6398 (11)	0.2322 (4)	0.5435 (11)	0.066 (3)	
H8	0.5966	0.2031	0.4881	0.080*	
C9	0.8003 (12)	0.2359 (5)	0.5862 (14)	0.091 (4)	
H9	0.8636	0.2109	0.5560	0.109*	
C10	0.8623 (15)	0.2778 (5)	0.6747 (14)	0.097 (5)	
H10	0.9683	0.2798	0.7079	0.116*	
C11	0.7701 (16)	0.3158 (5)	0.7133 (14)	0.094 (4)	
H11	0.8128	0.3446	0.7699	0.113*	
C12	0.6122 (14)	0.3116 (5)	0.6681 (11)	0.079 (3)	
H12	0.5495	0.3373	0.6969	0.094*	
C13	0.3026 (11)	0.2115 (3)	0.5086 (10)	0.055 (2)	
C14	0.2723 (13)	0.1847 (4)	0.6156 (12)	0.074 (3)	
H14	0.2993	0.2012	0.6990	0.089*	
C15	0.2004 (14)	0.1323 (4)	0.6000 (16)	0.088 (4)	
H15	0.1840	0.1135	0.6738	0.105*	
C16	0.1551 (14)	0.1091 (5)	0.4782 (18)	0.095 (4)	
H16	0.1010	0.0757	0.4681	0.114*	
C17	0.1888 (18)	0.1347 (6)	0.3678 (16)	0.116 (6)	
H17	0.1614	0.1184	0.2842	0.139*	
C18	0.2659 (17)	0.1861 (5)	0.3880 (12)	0.091 (4)	
H18	0.2926	0.2034	0.3164	0.109*	
C19	−0.4077 (11)	0.4192 (4)	0.1718 (10)	0.065 (3)	
H19A	−0.5148	0.4304	0.1460	0.078*	
H19B	−0.4058	0.3789	0.1771	0.078*	
C20	−0.3648 (13)	0.5048 (4)	0.3059 (11)	0.074 (3)	
H20A	−0.4723	0.5131	0.2740	0.111*	
H20B	−0.3078	0.5218	0.2488	0.111*	
H20C	−0.3294	0.5191	0.3943	0.111*	
C21	−0.4312 (12)	0.4165 (5)	0.3995 (10)	0.071 (3)	
H21A	−0.5382	0.4256	0.3684	0.107*	
H21B	−0.3936	0.4313	0.4872	0.107*	
H21C	−0.4189	0.3769	0.4015	0.107*	

### (III) N-(6,6-Diphenylhex-5-en-1-yl)-N-iodomethyl-N,N-dimethylammonium iodide Atomic displacement parameters (Å^2^)

**Table:**

	*U*^11^	*U*^22^	*U*^33^	*U*^12^	*U*^13^	*U*^23^
I1	0.0559 (4)	0.0659 (4)	0.0605 (4)	0.0039 (3)	0.0202 (3)	0.0118 (3)
I2	0.0586 (4)	0.0618 (4)	0.0632 (4)	0.0015 (3)	0.0228 (3)	0.0043 (3)
N1	0.041 (4)	0.081 (6)	0.046 (4)	0.002 (4)	0.012 (4)	0.020 (4)
C1	0.045 (5)	0.078 (7)	0.069 (6)	−0.006 (5)	0.015 (5)	0.014 (5)
C2	0.058 (6)	0.056 (6)	0.106 (9)	0.003 (5)	0.024 (6)	−0.001 (6)
C3	0.057 (6)	0.069 (6)	0.075 (7)	0.012 (5)	0.007 (6)	0.017 (6)
C4	0.054 (7)	0.065 (7)	0.158 (12)	0.006 (5)	0.036 (8)	0.023 (8)
C5	0.040 (5)	0.043 (5)	0.123 (9)	0.002 (4)	0.022 (6)	0.012 (6)
C6	0.047 (5)	0.038 (5)	0.082 (7)	0.005 (4)	0.019 (5)	0.010 (5)
C7	0.052 (6)	0.038 (5)	0.069 (6)	−0.002 (4)	0.019 (5)	0.010 (4)
C8	0.051 (6)	0.055 (6)	0.090 (7)	0.004 (5)	0.009 (6)	−0.006 (5)
C9	0.051 (7)	0.083 (8)	0.140 (11)	0.017 (6)	0.026 (8)	0.022 (8)
C10	0.066 (8)	0.077 (8)	0.129 (11)	−0.028 (7)	−0.014 (8)	0.022 (8)
C11	0.100 (10)	0.054 (7)	0.116 (11)	−0.008 (7)	−0.001 (9)	−0.007 (7)
C12	0.075 (8)	0.074 (7)	0.087 (8)	−0.007 (6)	0.019 (7)	−0.002 (7)
C13	0.056 (6)	0.040 (5)	0.067 (6)	0.012 (4)	0.011 (5)	0.014 (5)
C14	0.079 (8)	0.053 (6)	0.101 (9)	−0.004 (6)	0.043 (7)	−0.012 (6)
C15	0.085 (9)	0.041 (6)	0.152 (13)	−0.009 (6)	0.058 (9)	0.011 (7)
C16	0.053 (7)	0.063 (7)	0.155 (14)	−0.001 (6)	−0.004 (9)	−0.005 (10)
C17	0.135 (14)	0.072 (9)	0.108 (11)	0.001 (9)	−0.038 (10)	−0.002 (8)
C18	0.116 (11)	0.069 (8)	0.075 (8)	−0.001 (8)	−0.002 (7)	0.001 (7)
C19	0.049 (6)	0.080 (7)	0.073 (7)	−0.005 (5)	0.029 (5)	0.024 (6)
C20	0.066 (7)	0.071 (7)	0.089 (8)	0.009 (6)	0.027 (6)	0.008 (6)
C21	0.056 (6)	0.091 (8)	0.072 (7)	−0.006 (6)	0.025 (6)	0.032 (6)

### (III) N-(6,6-Diphenylhex-5-en-1-yl)-N-iodomethyl-N,N-dimethylammonium iodide Geometric parameters (Å, º)

**Table:**

I1—C19	2.138 (9)	C9—C10	1.388 (17)
I1—I2^i^	3.5565 (9)	C9—H9	0.9300
N1—C19	1.489 (12)	C10—C11	1.353 (18)
N1—C1	1.494 (12)	C10—H10	0.9300
N1—C21	1.515 (11)	C11—C12	1.383 (16)
N1—C20	1.537 (12)	C11—H11	0.9300
C1—C2	1.477 (13)	C12—H12	0.9300
C1—H1A	0.9700	C13—C18	1.360 (14)
C1—H1B	0.9700	C13—C14	1.365 (14)
C2—C3	1.543 (13)	C14—C15	1.407 (14)
C2—H2A	0.9700	C14—H14	0.9300
C2—H2B	0.9700	C15—C16	1.353 (18)
C3—C4	1.510 (14)	C15—H15	0.9300
C3—H3A	0.9700	C16—C17	1.39 (2)
C3—H3B	0.9700	C16—H16	0.9300
C4—C5	1.514 (14)	C17—C18	1.409 (17)
C4—H4A	0.9700	C17—H17	0.9300
C4—H4B	0.9700	C18—H18	0.9300
C5—C6	1.323 (12)	C19—H19A	0.9700
C5—H5	0.9300	C19—H19B	0.9700
C6—C7	1.497 (12)	C20—H20A	0.9600
C6—C13	1.507 (12)	C20—H20B	0.9600
C7—C8	1.366 (12)	C20—H20C	0.9600
C7—C12	1.379 (14)	C21—H21A	0.9600
C8—C9	1.401 (14)	C21—H21B	0.9600
C8—H8	0.9300	C21—H21C	0.9600
			
C19—I1—I2^i^	171.6 (3)	C11—C10—C9	120.6 (11)
C19—N1—C1	116.9 (8)	C11—C10—H10	119.7
C19—N1—C21	107.4 (7)	C9—C10—H10	119.7
C1—N1—C21	109.1 (7)	C10—C11—C12	119.7 (12)
C19—N1—C20	109.1 (7)	C10—C11—H11	120.1
C1—N1—C20	106.3 (7)	C12—C11—H11	120.1
C21—N1—C20	107.8 (8)	C7—C12—C11	121.7 (12)
C2—C1—N1	114.8 (8)	C7—C12—H12	119.1
C2—C1—H1A	108.6	C11—C12—H12	119.1
N1—C1—H1A	108.6	C18—C13—C14	119.1 (9)
C2—C1—H1B	108.6	C18—C13—C6	122.5 (9)
N1—C1—H1B	108.6	C14—C13—C6	118.4 (9)
H1A—C1—H1B	107.5	C13—C14—C15	120.1 (12)
C1—C2—C3	108.2 (8)	C13—C14—H14	119.9
C1—C2—H2A	110.1	C15—C14—H14	119.9
C3—C2—H2A	110.1	C16—C15—C14	120.4 (12)
C1—C2—H2B	110.1	C16—C15—H15	119.8
C3—C2—H2B	110.1	C14—C15—H15	119.8
H2A—C2—H2B	108.4	C15—C16—C17	120.6 (11)
C4—C3—C2	114.0 (9)	C15—C16—H16	119.7
C4—C3—H3A	108.7	C17—C16—H16	119.7
C2—C3—H3A	108.7	C16—C17—C18	117.3 (13)
C4—C3—H3B	108.7	C16—C17—H17	121.3
C2—C3—H3B	108.7	C18—C17—H17	121.3
H3A—C3—H3B	107.6	C13—C18—C17	122.2 (13)
C3—C4—C5	112.1 (9)	C13—C18—H18	118.9
C3—C4—H4A	109.2	C17—C18—H18	118.9
C5—C4—H4A	109.2	N1—C19—I1	117.2 (6)
C3—C4—H4B	109.2	N1—C19—H19A	108.0
C5—C4—H4B	109.2	I1—C19—H19A	108.0
H4A—C4—H4B	107.9	N1—C19—H19B	108.0
C6—C5—C4	124.8 (9)	I1—C19—H19B	108.0
C6—C5—H5	117.6	H19A—C19—H19B	107.2
C4—C5—H5	117.6	N1—C20—H20A	109.5
C5—C6—C7	123.1 (8)	N1—C20—H20B	109.5
C5—C6—C13	120.7 (8)	H20A—C20—H20B	109.5
C7—C6—C13	116.2 (7)	N1—C20—H20C	109.5
C8—C7—C12	117.9 (10)	H20A—C20—H20C	109.5
C8—C7—C6	121.6 (9)	H20B—C20—H20C	109.5
C12—C7—C6	120.5 (9)	N1—C21—H21A	109.5
C7—C8—C9	121.6 (10)	N1—C21—H21B	109.5
C7—C8—H8	119.2	H21A—C21—H21B	109.5
C9—C8—H8	119.2	N1—C21—H21C	109.5
C10—C9—C8	118.4 (11)	H21A—C21—H21C	109.5
C10—C9—H9	120.8	H21B—C21—H21C	109.5
C8—C9—H9	120.8		
			
C19—N1—C1—C2	−55.7 (12)	C8—C7—C12—C11	1.8 (16)
C21—N1—C1—C2	66.3 (12)	C6—C7—C12—C11	−176.9 (10)
C20—N1—C1—C2	−177.7 (9)	C10—C11—C12—C7	−2 (2)
N1—C1—C2—C3	−174.9 (8)	C5—C6—C13—C18	78.5 (15)
C1—C2—C3—C4	−171.1 (10)	C7—C6—C13—C18	−98.8 (13)
C2—C3—C4—C5	175.7 (10)	C5—C6—C13—C14	−100.9 (13)
C3—C4—C5—C6	165.3 (12)	C7—C6—C13—C14	81.9 (12)
C4—C5—C6—C7	175.5 (11)	C18—C13—C14—C15	−1.6 (16)
C4—C5—C6—C13	−1.5 (18)	C6—C13—C14—C15	177.7 (9)
C5—C6—C7—C8	−140.0 (11)	C13—C14—C15—C16	−2.7 (18)
C13—C6—C7—C8	37.2 (13)	C14—C15—C16—C17	4.7 (19)
C5—C6—C7—C12	38.6 (15)	C15—C16—C17—C18	−2 (2)
C13—C6—C7—C12	−144.2 (10)	C14—C13—C18—C17	4.0 (19)
C12—C7—C8—C9	−2.7 (16)	C6—C13—C18—C17	−175.3 (11)
C6—C7—C8—C9	175.9 (10)	C16—C17—C18—C13	−2 (2)
C7—C8—C9—C10	3.5 (18)	C1—N1—C19—I1	−54.3 (10)
C8—C9—C10—C11	−3.4 (19)	C21—N1—C19—I1	−177.2 (7)
C9—C10—C11—C12	3 (2)	C20—N1—C19—I1	66.3 (9)

Symmetry code: (i) −*x*, −*y*+1, −*z*.
